# Sustainable Chitosan-Based Nanoparticles for Curcumin Delivery: Assessment of Chemical Integrity and Antibacterial Activity Against *Escherichia coli*

**DOI:** 10.1021/acsomega.6c07855

**Published:** 2026-09-08

**Authors:** Valentina Verdoliva, Viviana De Luca, Clemente Capasso, Stefania De Luca

**Affiliations:** † Department of Environmental, Biological and Pharmaceutical Sciences and Technologies, National Research Council (CNR), Institute of Crystallography, Via Vivaldi43, 81100 Caserta, Italy; ‡ Department of Biology, Agriculture and Food Sciences, National Research Council (CNR), Institute of Biosciences and Bioresources, Via P. Castellino, 111, 80131 Naples, Italy; ⊥ Department of Biomedical Sciences, Institute of Biostructures and Bioimaging, National Research Council (CNR), Via P. Castellino, 111, 80131 Naples, Italy

## Abstract

Curcumin (CUR) is a natural polyphenol with recognized antibacterial and antioxidant properties, whose biomedical application is limited by poor aqueous solubility, low bioavailability, and rapid chemical degradation under physiological conditions. In this study, green and sustainable chitosan-fatty acid nanoparticles were developed through a mild cosolvent evaporation strategy to improve CUR stability and facilitate antibacterial activity while avoiding harsh preparation conditions. The obtained nanosystem showed that CUR encapsulation and formation of stable nanosized particles are suitable for aqueous dispersion and biological applications, since curcumin was efficiently transported into the intracellular compartment of *Escherichia coli* *.* This was used as Gram-negative bacterium to investigate the antibacterial activity of encapsulated CUR. In addition, the chitosan-based carrier effectively preserved the chemical integrity of CUR both during nanoparticle preparation and under physiological conditions. The combined effects of sustainable formulation, improved CUR stability, and antibacterial activity highlight the potential of these chitosan-based nanoparticles as effective delivery systems for poorly soluble bioactive molecules. Overall, the proposed strategy represents an environmentally friendly platform for the development of advanced antimicrobial nanomaterials based on natural polymers.

## Graphical abstract

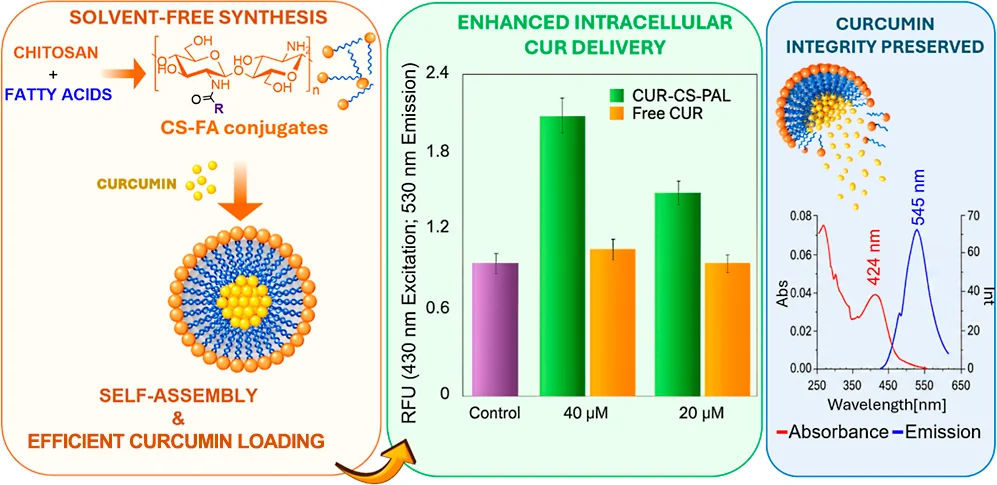

## 1 Introduction

Curcumin (CUR) is a polyphenolic bioactive compound derived from *Curcuma longa* and is widely recognized for its broad spectrum of biological activities, such as potent anti-inflammatory, antioxidant, antimicrobial, antiviral, anticancer, neuroprotective, and adjuvant therapeutic properties for several diseases (e.g., arthritis and metabolic syndrome) (Figure 1).^1–5^ The main hurdle to the pharmacological effectiveness of CUR is related, essentially, to its poor aqueous solubility and low bioavailability, mainly due to limited intestinal absorption, rapid metabolism, rapid systemic elimination, photodegradation, and chemical instability. A particularly important aspect of CUR instability concerns its behavior at physiological pH. Under neutral to alkaline conditions, CUR readily undergoes degradation through two main pathways: alkaline hydrolysis and autoxidation.^6,7^

**Figure 1:**
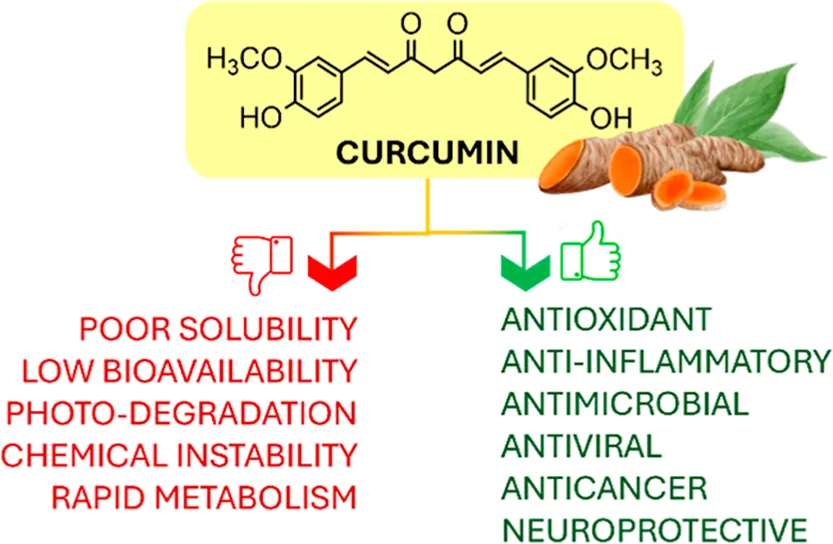
Biological activities and limitations of CUR.

**Table 1:** CUR Loading in CS-PAL, CS-OLE, and CS-LINO Nanoparticles Determined by RP-HPLC

	Peak area (a.u.)	μg/mL	μM
CS-PAL	1587.68	3.37	9.15
CS-OLE	248.41	0.53	1.43
CS-LINO	291.10	0.62	1.68

Note: The CUR concentrations reported in Table 1 correspond to the amount of encapsulated curcumin determined by RP-HPLC after purification and sample processing for formulation characterization. These values were used to calculate CUR loading. For biological experiments, lyophilized CUR-loaded nanoparticles were reconstituted in smaller volumes and adjusted to obtain the desired CUR-equivalent concentrations.

Biomaterial-based^8–13^ micelles are among the promising nanoformulations successfully employed to deliver CUR, providing prolonged circulation, better absorption, and prolonged half-life. Herein, we report the investigation of CUR encapsulated within a chitosan (CS)-based nanosystem as an antibacterial agent, with the final aim of ensuring the successful delivery of CUR through a stable and sustainable formulation.^14^

CUR has been widely reported to induce physical damage to the cell membranes of both Gram-positive and Gram-negative bacteria, thus causing bacterial cell death. In addition, CUR affects bacterial physiology by interfering with processes such as bacterial growth and biofilm formation.^15–18^ Several intracellular effects of CUR have been described in bacterial systems. In Gram-positive bacteria, CUR has been shown to interfere with cell division through interaction with FtsZ (filamenting temperature-sensitive mutant Z), a tubulin-like protein essential for cytokinesis.^19,20^ In Gram-negative bacteria, antibacterial activity is generally less pronounced and typically requires higher extracellular concentrations of CUR.^21,22^ This reduced efficacy is more commonly associated with limited intracellular availability of the compound.^23,24^ Since CUR exhibits poor aqueous solubility and poor chemical stability, its effective intracellular concentration in both Gram-positive and Gram-negative organisms is significantly limited.^25,26^ As a result, antibacterial activity may depend as much on compound delivery and intracellular availability as on molecular target interactions.^16,26^ In Gram-negative bacteria, the outer membrane further limits intracellular access, making uptake a key determinant of antibacterial efficacy.^27,28^ In this context, *Escherichia coli* serves as a relevant model organism,^29,30^ being also extensively characterized, while genetically and physiologically tractable. Accordingly, in the present study, *E. coli* was employed as a model system to investigate the intracellular biological activity of CUR delivered through the chitosan-based nanosystem. CS-palmitate (CS-PAL), CS-oleate (CS-OLE), and CS-linoleate (CS-LINO) were synthesized following a previously published solvent-free protocol in which microwave irradiation promotes the reaction between chitosan and natural fatty acids (FA).^14,31^ An optimized procedure for the formation of CS-based micelles with simultaneous encapsulation of CUR was then developed, and CS-PAL micellar aggregates exhibited the highest CUR loading efficiency. The resulting nanosystem was thoroughly characterized by dynamic light scattering (DLS), and its colloidal stability was monitored over time. Furthermore, the chemical integrity of curcumin following encapsulation and extraction from the nanoparticles was confirmed by UV–Vis and fluorescence spectroscopy. CUR-loaded CS-PAL nanoparticles were subsequently investigated by the fluorescence microscopy to monitor the intracellular localization of fluorescent compound in *E. coli* cells. The growth-modulating effects of the nanosystem encapsulating CUR were then investigated by treating *E. coli* with different concentrations of both empty CS-PAL nanoparticles and CUR-loaded nanoparticles. Because free CUR showed no detectable intracellular accumulation under the experimental conditions employed, subsequent antibacterial assays only focused on the nanoparticle formulation and its corresponding carrier controls. Minimum inhibitory concentration (MIC) and minimum bactericidal concentration (MBC) assays were also performed. The obtained results demonstrated that bacterial growth inhibition was mainly associated with the antibacterial activity of CUR released from the nanoparticles and internalized into bacterial cells.

## 2 Materials and Methods

### 2.1 Materials and Reagents

CS low molecular weight (50,000–190,000 Da; deacetylation >75%), palmitic anhydride, oleic acid, linoleic acid, DIC, K_2_CO_3_, and all solvents were purchased from Sigma-Aldrich (St. Louis, MO, USA). Spectrum Laboratories Spectra/Por 3 3.5 kDa MWCO Standard RC Dry Dialysis Kits were purchased from Thermo Fisher Scientific (Waltham, MA, USA); *E. coli* cells (biosafety level 1, Gram-negative) were used as a prokaryotic model system.^29^ Bacteria were grown in Mueller–Hinton (MH) medium under standard laboratory conditions at 37 °C with shaking until midexponential phase.

### 2.2 Synthesis

CS-FA conjugates were synthesized accordingly to the protocol recently published.^14^ Purified samples were recovered with a yield of approximately 30%–40%.

### 2.3 FT-IR Characterization

CS and CS-FA conjugates were characterized by FT-IR spectroscopy using the ATR accessory of a JASCO FT/IR-4100 spectrometer (JASCO Europe S.r.l., Cremella, Italy). Spectra were recorded in the 400–4000 cm^–1^ range with 16 scans and a resolution of 4 cm^–1^.

CS: ∼3355 cm^–1^ (O–H stretching), 3290 cm^–1^ (O–H/N–H stretching), ∼2923 cm^–1^ (C–H stretching), 1646 cm^–1^ (C=O stretching amide II of acetyl groups), 1571 cm^–1^ (NH bending amide II), 1373 cm^–1^ (CH_2_ bending), 1155 cm^–1^ and 1085 cm^–1^ (C–O stretching), and 898 cm^–1^ (β-glycosidic bond) in the fingerprint region.

CS-FA conjugates: ∼3355 cm^–1^ (O–H stretching), 3290 cm^–1^ (O–H/N–H stretching, 2923–2850 cm^–1^ (FA alkyl chains C–H stretching), 1671 cm^–1^ (C=O stretching amide II bond between CS-NH_2_ and FA–COOH), 1646 cm^–1^(C=O stretching amide II of acetyl groups), 1526 cm^–1^ (N–H bending amide II), 1373 cm^–1^ (CH_2_ bending), 1155 cm^–1^ and 1085 cm^–1^ (C–O stretching), and 898 cm^–1^ (β-glycosidic bond) in the fingerprint region.

### 2.4 Formulation and Characterization of CUR-Loaded CS-Fatty Acid Nanoparticles

CUR-loaded CS-FA nanoparticles were prepared by a cosolvent evaporation method using ethanol as organic phase.^32^

CS-FA conjugates (3 mg) were dissolved in 0.9% (w/v) NaCl aqueous solution (3 mL) under magnetic stirring. CUR (0.5 mg) was dissolved in EtOH (0.5 mL). The amounts of CUR and EtOH were optimized in preliminary experiments to achieve complete drug dissolution and maintain colloidal stability after aqueous phase incorporation. CUR solution was added dropwise to the CS-FA aqueous solution under magnetic stirring (800–1000 rpm), and the obtained dispersion was sonicated for 5 min to enhance mixing.

The resulting suspension was emulsified under gentle stirring overnight at room temperature and protected from light to prevent photodegradation. This allowed the organic solvent evaporation and concomitant nanoparticles formation into the aqueous layer. Residual EtOH was slowly removed by using a flow of compressed air for about 1 h.

The aqueous phase was subsequently centrifuged at 13,000 rpm for 10 min to eliminate the excess of insoluble CUR, then a size-exclusion chromatography (Sephadex G50 column, GE Healthcare, Uppsala, Sweden) was performed to isolate the fraction of formed nanoparticles.

Subsequently, the purified nanoparticles were characterized in triplicate by dynamic light scattering (DLS) using a Zetasizer Pro instrument ZETASIZER PRO (Malvern Panalytical, Malvern, UK; Almelo, The Netherlands). Measurements were performed at 25.0 ± 0.1 °C using ZEN0040-low volume disposable cuvettes (40–45 μL) and water as the dispersant.

### 2.5 Encapsulated CUR Quantification

The encapsulation efficiency of CUR within CS–FA nanoparticles was determined by HPLC analysis and referring to the developed calibration curve.

#### 2.5.1 Calibration Curve

A CUR standard stock solution was prepared by dissolving 1 mg of free CUR in 5.0 mL acetonitrile (CH_3_CN), obtaining a final concentration of 200 μg/mL. Calibration standards were prepared by a serial dilution of the stock solution with acetonitrile to obtain concentrations respectively of 100, 50, 25, and 13 μg/mL. The resulting standard solutions were analyzed by RP-HPLC, and the integrated peak areas obtained for each of them were plotted against the related concentrations to generate the calibration curve necessary for CUR quantification.

#### 2.5.2 RP-HPLC Analysis

A volume of 400 μL of the purified nanoparticle water solutions (*V*_tot_ = 4.4 mL) was lyophilized and subsequently dissolved in 1.5 mL ethanol (EtOH). The suspension was sonicated for 30 min to disrupt the micellar structure and promote the release of the encapsulated CUR. The sample was then centrifuged at 13,000 × g for 7 min to remove insoluble polysaccharide conjugate fraction, and the resulting yellow supernatant was collected. Ethanol was completely removed under reduced pressure using a rotary evaporator, and the dried residue was reconstituted in 100 μL of acetonitrile.

The samples were analyzed using an Agilent 1290 Infinity LC system coupled to an Agilent 6230 TOF LC/MS system (Agilent Technologies, Cernusco sul Naviglio, Italy). Chromatographic separation was carried out on a Phenomenex Jupiter C18 column (3 μm, 300 Å, 150 × 2.0 mm) at a flow rate of 0.2 mL·min^–1^. The mobile phase consisted of H_2_O containing 0.1% TFA (A) and CH_3_CN containing 0.1% TFA (B), using a linear gradient from 50% to 90% B over 13 min. Detection was performed at 420 nm. Quantification of CUR was achieved by injection of 10 μL of each sample (*V*_tot_ = 100 μL) and integration of the obtained chromatographic peak areas. The amount of CUR (μL/mL) was estimated by using the calibration curve (Figure 2).

**Figure 2:**
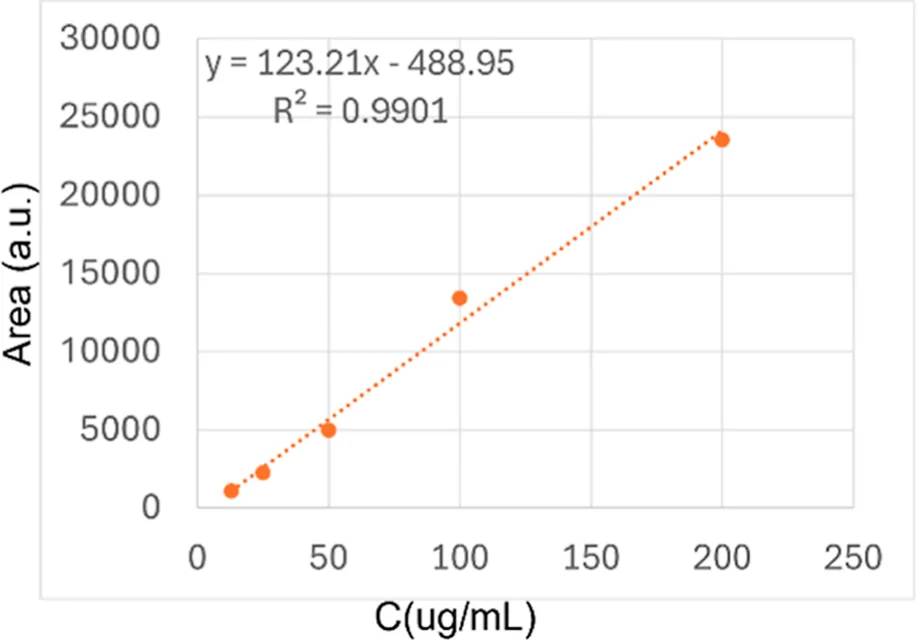
RP-HPLC calibration curve for CUR obtained by plotting the integrated chromatographic peak area (a.u.) versus curcumin concentration (μg/mL). Data are expressed as mean ± SD of three independent measurements (*n* = 3). The linear regression equation (*R* ^2^ = 0.9901) was used for quantitative determination of encapsulated curcumin.

### 2.6 Spectroscopic Characterization of the Extracted CUR from Nanoparticles

400 μL of the nanoparticle water solutions (*V*_tot_ = 4.4 mL) was lyophilized and subsequently dissolved in 1.5 mL of EtOH. The suspension was sonicated for 30 min to disrupt the micellar structure and promote the release of the encapsulated CUR. The sample was then centrifuged at 13,000 × g for 7 min to remove the insoluble polysaccharide conjugate fraction, and the resulting yellow supernatant was analyzed by UV–vis and Fluorescence spectroscopy.

Jasco V-730 spectrophotometer equipped with an ETCS-761 temperature controller was employed. Spectra were recorded in the 230–600 nm range at room temperature using 500 μL quartz cuvettes, with blank correction. The experimental parameters were set as follows: scan speed of 200 nm·min^–1^, data interval of 0.2 nm, response time of 0.24 s, continuous scan mode, and bandwidth of 1.0 nm. The presence of the characteristic absorption maximum of CUR at λ = 424 nm was observed.

Fluorescence spectra were recorded at room temperature using a JASCO FP-8350 spectrofluorimeter equipped with an ETC-115 temperature controller and a 1.0 cm quartz cuvette, with blank correction. The experimental parameters were set as follows: scan speed of 200 nm·min^–1^, data interval of 0.5 nm, and medium sensitivity.

Samples were excited at 430 nm, and emission spectra were collected in the 440–650 nm range. The characteristic fluorescence emission maximum of CUR was observed at λ = 545 nm.

### 2.7 In Vitro CUR Release Study

To investigate CUR release, lyophilized CUR-loaded CS-PAL nanoparticles were reconstituted in 2 mL Milli-Q water and transferred into a dialysis membrane (MWCO 3.5 kDa). The dialysis bag was immersed in a 50 mL Falcon tube containing phosphate buffer solution (0.1 M, pH = 7.4) as release medium and maintained under magnetic stirring for up to 72 h.

At predetermined time intervals (0.30, 1, 2, 4, 8, 24, 36, 48, 72 h), 2 mL aliquots of the release medium were withdrawn and analyzed by UV–vis spectroscopy at λ = 430 nm. After each withdrawal, an equal volume of fresh phosphate buffer was added to maintain sink conditions.

### 2.8 Fluorescence Microscopy Analysis of up Taken CUR in *E. coli* Bacteria

*E. coli* cells were harvested at midexponential growth phase, collected by centrifugation, and resuspended in physiological saline solution (0.9% NaCl). Bacterial suspensions were adjusted to 1 × 10^5^ cells and incubated with either free CUR (40 μM obtained from a stock solution in DMSO and diluted in physiological saline, ensuring that the final DMSO content did not exceed 1%) or CUR-loaded CS-PAL nanoparticles (40 μM of encapsulated CUR) in a final volume of 200 μL. Incubations were carried out for 30 min at 37 °C under constant shaking at 200 rpm. Untreated cells were processed in parallel as negative controls. The incubation time and experimental conditions were selected to enable visualization of intracellular fluorescence while minimizing cellular stress and preserving membrane integrity, as inferred from the absence of acute growth defects in downstream assays. Following incubation, cells were collected by centrifugation, washed three times with physiological saline solution to remove extracellular CUR or nanoparticles, and finally resuspended in saline solution. Extracellular fluorescence was quenched by addition of trypan blue following the final washing step and prior to imaging.^33,34^ Trypan blue was used to suppress fluorescence arising from extracellular or surface-exposed fluorophores, while preserving intracellular fluorescence signals in cells with intact membranes. An aliquot of each sample was then withdrawn and directly subjected to fluorescence microscopy analysis. Imaging was performed using an EVOS M5000 fluorescence microscope (Life Technologies) equipped with a GFP filter cube (482/25 nm Excitation; 524/24 nm Emission). Fluorescence excitation and emission settings were selected to enable direct and internally consistent comparison across all experimental conditions rather than to maximize detection sensitivity for specific CUR microenvironments. Observations were conducted using an Olympus 100× Objective (semiapo, 0.95NA/0.2WD, with the correction collar), which provides optimal spatial resolution for visualization of prokaryotic cells. Bright-field images were collected in transmitted-light mode and were not acquired using phase-contrast optics. For each condition, brightfield, fluorescence, and merged images were acquired using identical acquisition parameters. Images shown are representative of three independent experiments.

### 2.9 Intracellular CUR Quantification in Bacterial Lysates

To quantitatively assess intracellular accumulation of CUR, *E. coli* cells treated with either CUR-loaded CS-PAL or free CUR were subjected to cell lysis followed by fluorescence measurement. Briefly, cells were harvested after treatment, collected by centrifugation, and washed three times with physiological saline solution to remove extracellular and surface-associated material. After the final wash, cell pellets were resuspended in physiological saline at the same volume as the initial sample. Cell lysis was achieved by repeated freeze–thaw cycles to ensure disruption of bacterial membranes without chemical detergents. Following lysis, samples were centrifuged to remove debris, and cleared supernatants were collected. Fluorescence was measured using a Tecan Spark multimode microplate reader (430 nm excitation; 530 nm emission). Untreated cells and cells treated with free CUR were processed as controls.

### 2.10 Bacterial Growth Modulation Assays

To evaluate the effect of CUR-loaded CS-PAL nanoparticles, *E. coli* cells were grown under standard conditions until the desired optical density was reached. For early phase treatment, cultures were adjusted to approximately 1 × 10^5^ cells/mL, corresponding to an OD_600_ of ∼0.15. For exponential-phase treatment, nanoparticles were added at OD_600_ ∼0.6. The CUR content of the nanoparticle formulation was previously quantified by RP-HPLC. Prior to biological experiments, purified CUR-loaded CS-PAL nanoparticles were lyophilized and reconstituted in reduced volumes, allowing preparation of suspensions with the desired CUR-equivalent concentrations. CUR-loaded CS-PAL nanoparticles were added at final CUR concentrations of 20 μM or 40 μM. Empty CS-PAL nanoparticles and untreated cells were used as controls. Cultures were incubated at 37 °C with shaking, and growth was monitored over time by measuring OD_600_. All experiments were performed in independent biological replicates, and growth curves are reported as mean OD_600_ values with associated variability.

### 2.11 Colony-forming Unit (CFU) Assay

Colony-forming unit (CFU) assessment was performed under conditions matching the growth modulation assays. In detail, *E. coli* cultures were treated with CUR-loaded CS-PAL nanoparticles (20 μM and 40 μM) or empty CS-PAL nanoparticles and then tested after incubation. Aliquots were serially diluted in sterile physiological saline and plated onto drug-free LB agar plates. Plates were incubated at 37 °C for 24 h, after which colonies were examined to evaluate bacterial recovery. The presence of viable colonies was qualitatively assessed and considered together with MIC and MBC determinations. CFU assessment was used as a complementary indicator of bacterial viability to support the distinction between bacteriostatic and bactericidal effects. All measurements were performed in independent biological replicates and analyzed in parallel with OD_600_ growth data.

### 2.12 Minimum Inhibitory Concentration (MIC) and Minimum Bactericidal Concentration (MBC) Evaluation

The antibacterial activity of CUR-loaded CS-PAL nanoparticles was assessed by determining MIC and MBC using a broth microdilution assay in accordance with CLSI guidelines. Prior to antibacterial testing, purified CUR-loaded CS-PAL nanoparticles were lyophilized and reconstituted in reduced volumes to obtain the desired CUR-equivalent concentrations. Exponentially growing *E. coli* cultures were diluted to approximately 1 × 10^5^ CFU/mL and exposed to CUR-CS-PAL at concentrations ranging from 5 to 160 μM (CUR-equivalent concentrations calculated from the RP-HPLC-determined CUR loading). Controls included untreated cells and empty nanoparticles. After 24 h of incubation, growth was assessed visually and by OD_600_ measurement using a Tecan Spark multimode microplate reader. MIC was defined as the lowest concentration preventing visible growth. For MBC determination, aliquots from wells showing no growth were plated on drug-free agar and incubated for 24 h. MBC was defined as the lowest concentration resulting in no colony formation (≥99.9% reduction in viable cells). All experiments were performed in independent biological replicates.

### 2.13 Statistical Analysis

Data are presented as mean ± standard deviation (SD) from three independent biological experiments (*n* = 3). Intracellular curcumin fluorescence measurements (Figure 8) were analyzed by one-way ANOVA followed by Tukey’s multiple-comparison test. Bacterial growth curves (Figure 9) were analyzed using two-way repeated-measures ANOVA, considering treatment and time as factors, followed by Tukey’s multiple-comparison test. Differences were considered statistically significant at *p* < 0.05.

**Figure 3:**
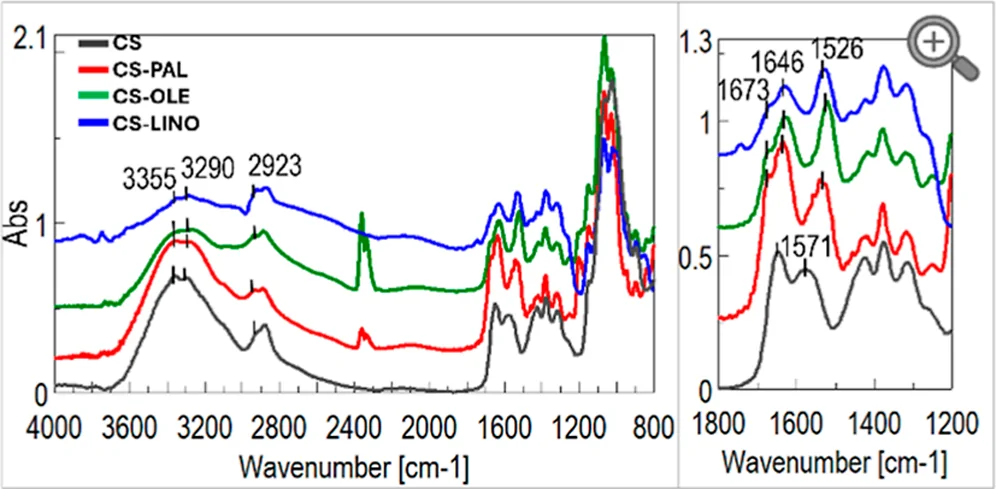
Normalized FT-IR spectra of CS-FA conjugates.

**Figure 4:**
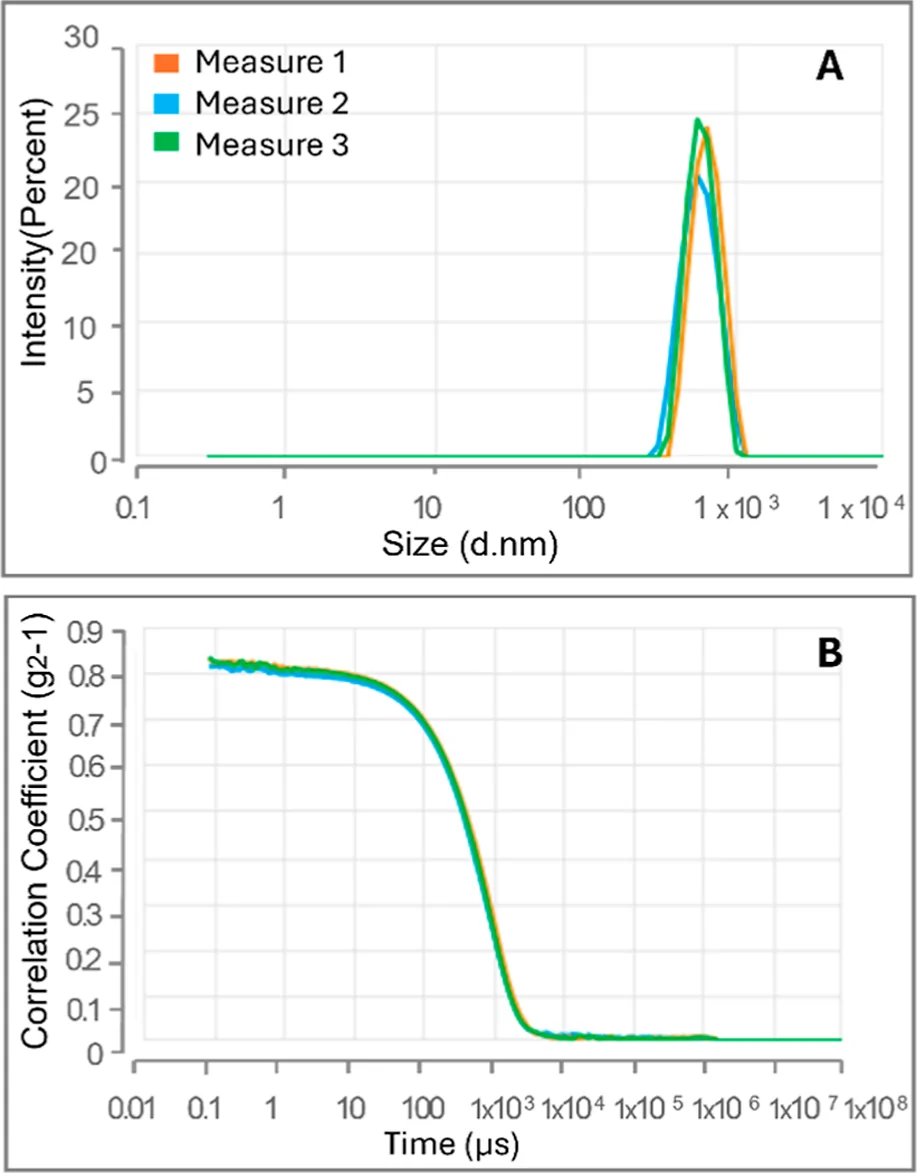
Size distribution (A) and intensity correlation functions of CUR- loaded CS-PAL nanoparticles (B).

**Figure 5:**
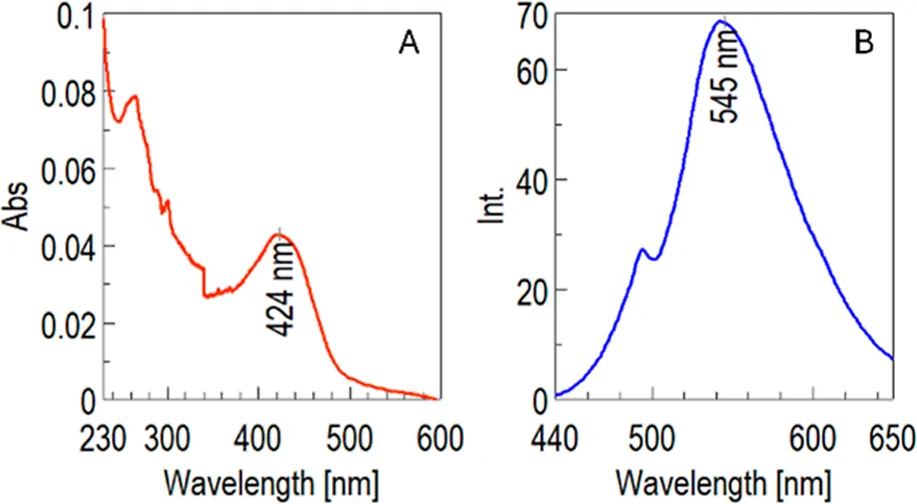
UV–vis absorption (A) and fluorescence emission (B) spectra of CUR extracted from CS-PAL micelles.

**Figure 6:**
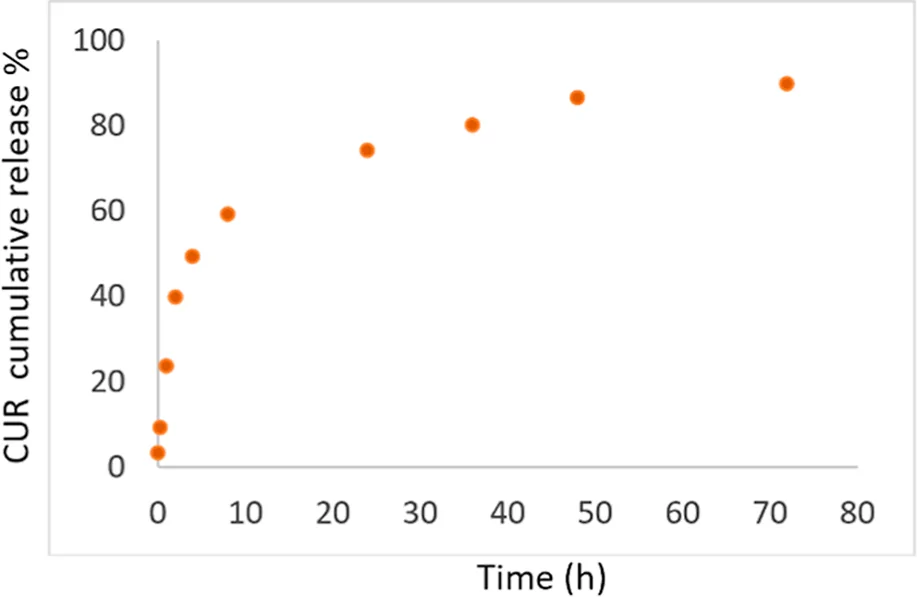
In Vitro CUR release study.

**Figure 7:**
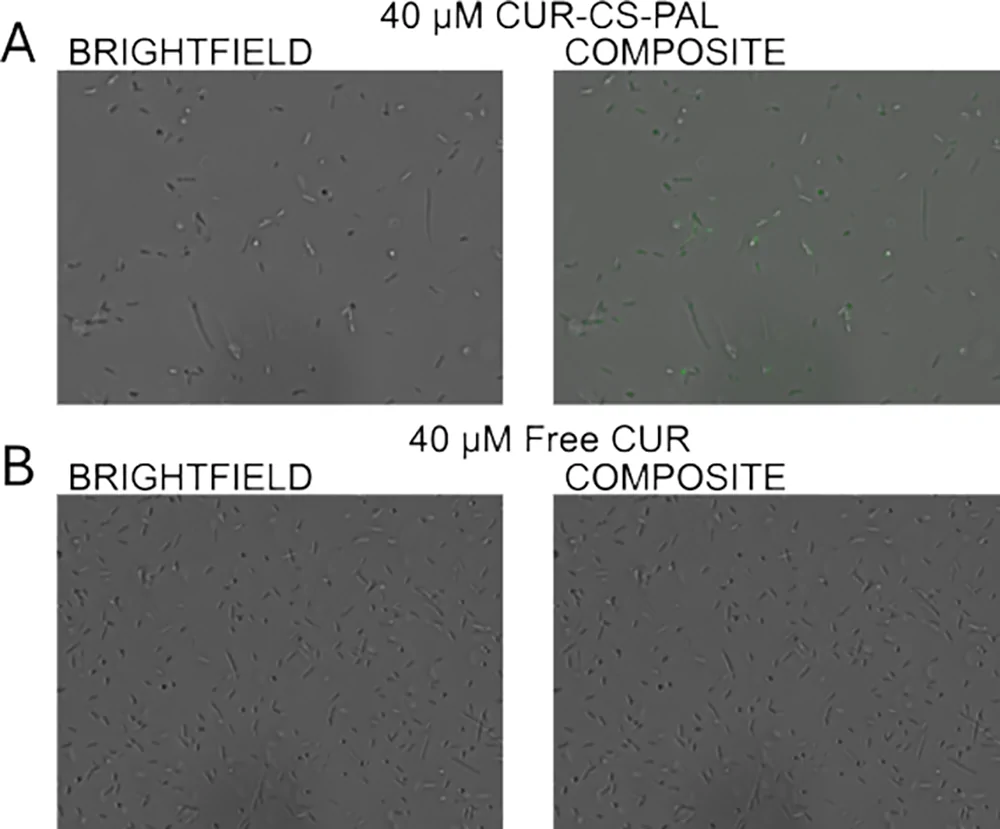
Representative brightfield and composite images of *E. coli* cells incubated with CUR-loaded CS-PAL (A) and free CUR (B). Merged images were selected to simultaneously visualize bacterial morphology and intracellular fluorescence localization. Quantitative assessment of intracellular CUR accumulation is provided independently in Figure 8. Brightfield images were acquired in transmitted-light mode and are not phase-contrast images. Scale bar = 2 μm.

**Figure 8:**
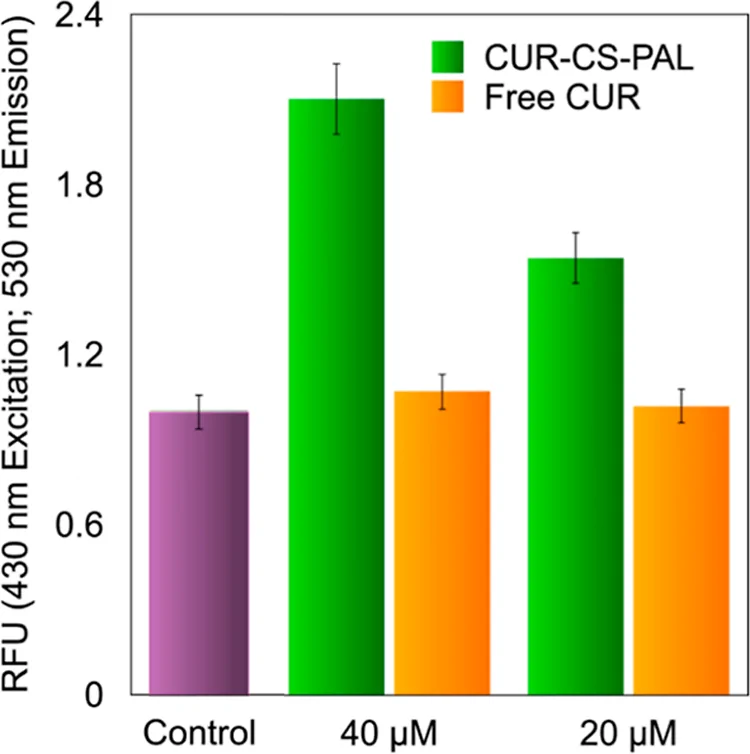
Quantitative analysis of intracellular CUR fluorescence. Relative fluorescence units (RFU) measured in *E. coli* cell lysates after treatment with free CUR or CUR-loaded CS-PAL nanoparticles. Data are reported as mean ± SD from three independent biological experiments (*n* = 3). Statistical significance was assessed by one-way ANOVA followed by Tukey’s multiple-comparison test.

**Figure 9:**
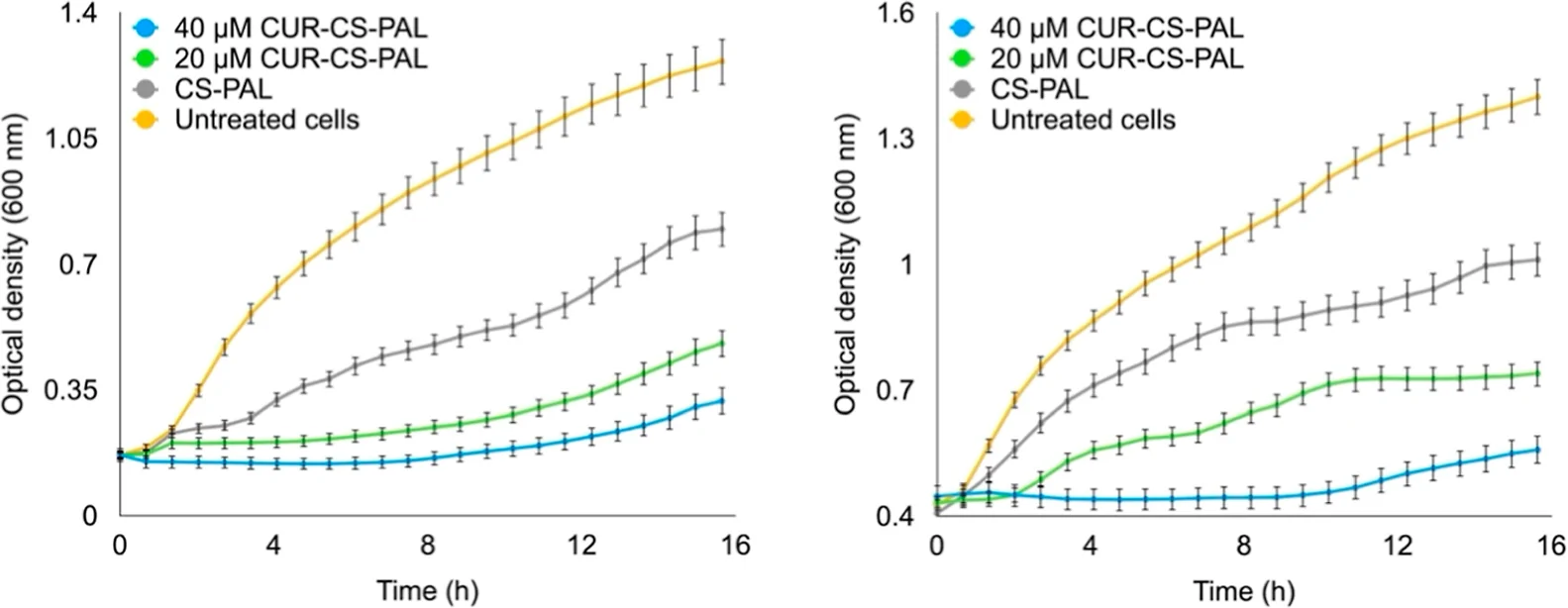
*E. coli* growth upon treatment with CUR-loaded CS-PAL and empty CS-PAL. Bacterial growth was monitored at OD_600_ measurements following nanoparticle addition during either the early growth phase (left) or the exponential growth phase (right). In the left panel, nanoparticles were added at the seeding/early growth stage (initial OD_600_ ≈ 0.15), whereas in the right panel nanoparticles were added after cultures had reached the exponential phase (initial OD_600_ ≈ 0.6). In both cases, growth monitoring started immediately after nanoparticle addition. Data represent mean ± SD from three independent biological experiments (*n* = 3). Statistical significance was assessed by two-way repeated-measures ANOVA followed by Tukey’s multiple-comparison test.

## 3 Results and Discussion

CS-FA conjugates were synthesized according to the previously published procedure.^14^ A MW-assisted *N*-acylation of CS with palmitic, oleic, and linoleic fatty acid anhydrides under solvent-free conditions was performed. Oleic and linoleic anhydrides were prepared following the previously described MW-assisted protocol, whereas palmitic anhydride was commercially available and used without further purification (Scheme 1).^35–37^

**Scheme 1:**
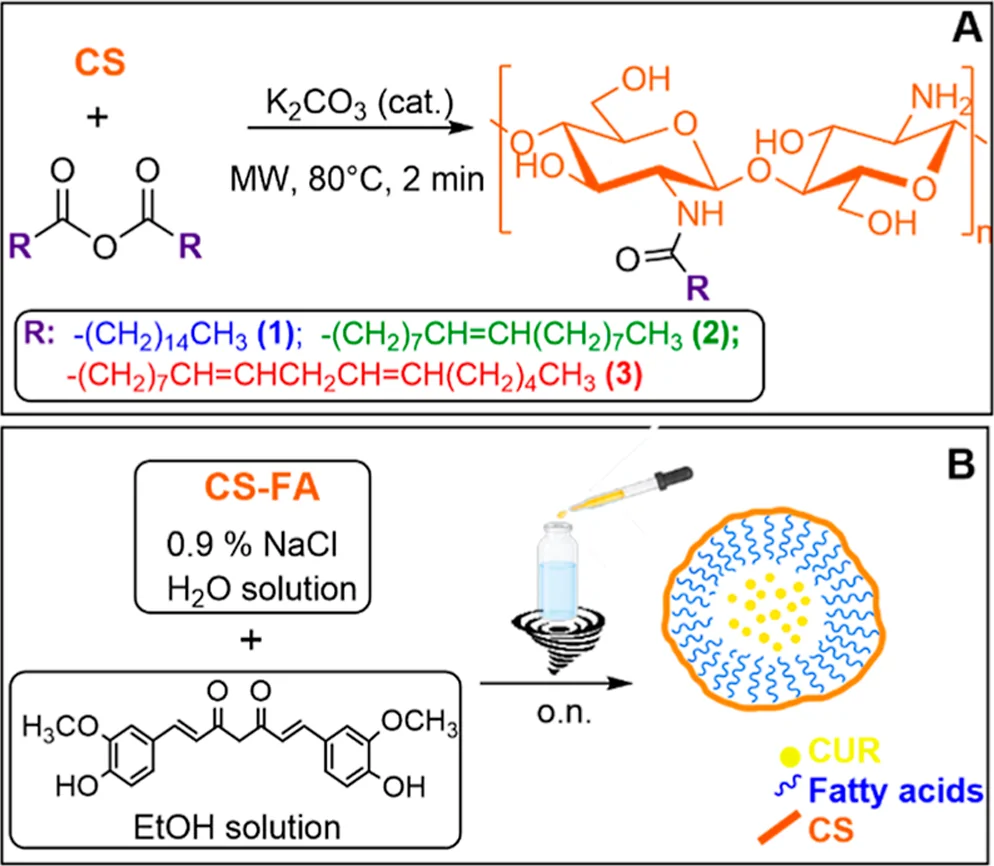
MW-Assisted Synthetic Strategy for CS-FA Conjugates (Panel A) and Protocol to Prepare CUR-Loaded CS-FA Nanoparticles (Panel B)

FT-IR characterization confirmed the successful conjugation reaction through the formation of amide bonds between chitosan and fatty acids.

In addition to the characteristic bands of the polysaccharide, including the broad absorption band at 3355–3290 cm^–1^ attributed to O–H and N–H stretching vibrations, the CS-FA conjugates exhibited a new band. This appeared as a shoulder at approximately 1673 cm^–1^ and was assigned to the C=O stretching vibration of the newly formed amide bond between CS-NH_2_ and FA-COOH groups. In contrast, the band at 1646 cm^–1^ was attributed to the C=O stretching vibration of the amide groups of residual acetyl moieties. Furthermore, a prominent band at 1526 cm^–1^, corresponding to N–H bending vibrations (amide II), further confirmed the formation of the new amide bond (Figure 3).

Concerning the drug formulation procedure, a protocol involving CUR encapsulation during the formation of CS-FA micelles was adopted. In previous formulations, the emulsion-evaporation method was employed using an organic solvent immiscible with water.^38,39^ In the present study, a modified strategy was developed to optimize and enhance CUR loading within the nanoparticles.

As shown in Scheme 1 (Panel B), a co-solvent evaporation procedure was employed,^32^ which consists of dissolving the drug in an organic solvent miscible with water. CUR was dissolved in the minimum volume of EtOH and added dropwise to an aqueous solution (0.9% NaCl) containing the CS-FA conjugate. The resulting mixture was sonicated and subsequently shaken overnight to allow evaporation of the ethanol fraction. After 15 h, a flow of compressed air was applied for 1 h to ensure complete removal of EtOH. The process was carried out slowly and resulted in cooling of the reaction vessel; both conditions favored the incorporation of CUR into the micelles rather than its precipitation in the aqueous phase, where CUR is essentially insoluble.

The obtained nanoparticles were characterized by dynamic light scattering (DLS) to evaluate their mean hydrodynamic diameter, polydispersity index (PDI), and colloidal stability. Measurements were performed in an aqueous solution (0.9% NaCl) and the size distribution profile of the nanosystems was assessed to evaluate the homogeneity of the nanoparticle population. All data collected are shown in Table 2.

**Table 2:** Characteristic Parameters of CUR-Loaded CS-FA Micelles

	mean diameter (nm)	PDI	δ(mV)
CUR-CS-PAL	546.9 ± 4.89	0.16 ± 0.018	18.53 ± 1.60
CUR-CS-OLE	281.4 ± 4.73	0.26 ± 0.035	16.83 ± 1.49
CUR-CS-LINO	488.9 ± 57.6	0.24 ± 0.067	17.02 ± 1.22

The mean diameters of CUR-loaded CS-FA nanoparticles ranged from 280 to 550 nm, depending on the specific fatty acid conjugate, which were larger than those of the empty nanoparticles.^14^ Next, to evaluate loading efficiency, the CUR content (final concentration) in the CS-FA micelles was estimated using a calibration curve obtained from a series of known standard concentrations. After disrupting the aggregates in EtOH via sonication, the released CUR was recovered by centrifugation, and the absorbance was measured at 420 nm. The final concentration was then calculated by comparing the peak area of the signal obtained against the calibration curve.

The highest content of CUR was found within CS-PAL nanoparticles (∼9 μM), also supported by the largest hydrodynamic diameter estimate by DLS analysis (Figure 4).

CUR-loaded CS-PAL nanoparticles were selected as the most promising drug delivery system based on their ability to incorporate a high amount of CUR. To ensure their suitability for subsequent biological applications, these micelles were disrupted in ethanol and the released curcumin was carefully studied. Specifically, the CUR extracted from the CS-PAL nanosystem was structurally analyzed by recording its UV–vis and fluorescence spectra. The characteristic absorbance/emission bands ensured the integrity of the CUR chemical structure, and no hydrolytic degradation occurred (Figure 5). This aspect is particularly relevant when a novel carrier system is developed for the delivery of a sensitive molecule such as CUR. Given the limited chemical stability of curcumin under physiological conditions, its encapsulation within a nanocarrier is essential not only to facilitate its delivery but also to prevent premature degradation, thus improving its stability, bioavailability, and overall therapeutic efficacy.

The stability of the developed nanocarrier of CUR was investigated performing a drug release experiment of CUR from the CS-PAL nanoparticles over 72 h at room temperature. Three different samples were prepared in a phosphate buffer (C = 0.10 M, pH = 7.4). A progressive CUR release, estimated by reading the drug absorbance at 420 nm, over time was observed until saturation was reached after ∼35 h. Figure 6 displays the percentage of drug released into the solution as a function of time for a representative experiment. In particular, the cumulative drug released was estimated to be around 86.4% (SD = ± 3.2) after 72 h.

### 3.1 Fluorescence Microscopy Analysis of *E. coli* Treated with CUR-Loaded CS-PAL Nanoparticles and Free CUR

CUR-loaded CS-PAL nanoparticles were evaluated for their ability to promote intracellular CUR delivery in *E. coli*. To this aim, the intrinsic fluorescence of CUR inside bacterial cells was analyzed by fluorescence microscopy. *E. coli* cells were incubated with either free CUR or CUR encapsulated in CS-PAL nanoparticles, while untreated cells were used as controls. Incubation of *E. coli* cells with 40 μM of CUR encapsulated in CS-PAL nanoparticles for 30 min at 37 °C resulted in a clearly detectable intracellular fluorescence signal (Figure 7A). The fluorescence signal appeared uniformly distributed within the bacterial cells and was completely absent in untreated control cells, providing direct visual evidence of efficient intracellular accumulation of CUR. Importantly, incubation under these conditions did not result in overt cell lysis or acute growth impairment. In contrast, incubation of *E. coli* cells with 40 μM free curcumin, under the same experimental conditions, did not result in detectable intracellular fluorescence (Figure 7B), indicating the absence of detectable intracellular accumulation of CUR under these conditions.

The fluorescence signal observed for *E. coli* treated with CUR-loaded CS-PAL nanoparticles was preserved upon treatment with trypan blue,^33,34^ indicating that the detected signal does not derive from surface-bound CUR or nanoparticles. No fluorescence was detected in cells treated with free CUR under any condition, further excluding a surface-associated contribution. All samples were imaged under identical acquisition settings, ensuring that differences in fluorescence signal reflect differences in cellular access rather than detection bias. Additionally, CS-based nanoparticles are characterized by a positive surface charge due to protonated amino groups, whereas the surface of *E. coli* cells is overall negatively charged, largely because of lipopolysaccharides and other anionic components of the outer membrane.^14,40^ This charge complementarity is likely crucial to promote nanoparticle–cell association. Such electrostatic interactions may therefore contribute to overcoming the permeability barrier of Gram-negative bacteria and favor subsequent intracellular delivery of CUR. The obtained results indicated that CS-PAL nanoparticles primarily act by enabling intracellular access of CUR rather than altering its intrinsic mode of action. These findings suggest that improved intracellular availability may contribute to the antibacterial effects observed for the nanoparticle formulation.^16,27^

### 3.2 Quantitative Analysis of Intracellular CUR Accumulation on *E. coli* Lysates

To further verify that CUR delivered by CS-PAL was effectively localized inside *E. coli* cells and not merely associated with the cell surface, bacterial cells were treated with the same concentration (20 μM and 40 μM) of either CUR-loaded CS-PAL nanoparticles or free CUR, extensively washed to remove extracellular and weakly surface-associated compounds, and subsequently lysed through repeated freeze–thaw cycles. After centrifugation, the fluorescence of the recovered supernatants was measured, and a significant increase in fluorescence signal was observed for cells treated with CUR-loaded CS-PAL nanoparticles compared with both free CUR-treated cells and untreated control cells (Figure 8).

It is worth noting that, while lysates from cells incubated with free CUR at concentrations of 20 and 40 μM exhibited fluorescence signals comparable to those of untreated control cells, lysates from cells treated with CUR-loaded CS-PAL nanoparticles showed a concentration-dependent increase in fluorescence intensity, indicating enhanced cellular uptake of CUR mediated by the nanoparticle delivery system. The apparent discrepancy between the qualitative microscopy observations (Figure 7) and the magnitude of the fluorescence increase measured in bacterial lysates (Figure 8) likely reflects the different sensitivities of the two techniques. While fluorescence microscopy requires local fluorescence intensity to exceed the visualization threshold in individual cells, fluorescence measurements on lysates integrate the signal from the entire bacterial population and can therefore detect lower levels of cell-associated CUR. Accordingly, fluorescence signals measurable in lysates may not necessarily produce a clearly visible signal in microscopy images acquired under identical experimental settings. Fluorescence measured in cell lysates reflects CUR associated with the cellular fraction after removal of extracellular material. Although a minor contribution from tightly membrane-associated compound cannot be entirely excluded, the data strongly support intracellular accumulation as the predominant source of the signal. This biochemical analysis provides an independent and quantitative confirmation of the microscopy data shown in Figure 7, demonstrating that curcumin delivered via CS-PAL nanoparticles is internalized beyond the *E. coli* membrane surface. Given the absence of detectable intracellular accumulation of free CUR under the experimental conditions employed in this study, subsequent antibacterial activity studies, including growth inhibition and MIC and MBC determinations, were focused on CUR-loaded CS-PAL nanoparticles.

### 3.3 Effects of CUR-Loaded CS-PAL Nanoparticle on *E. coli* Growth

Next, the effects of the intracellularly delivered CUR via CS-PAL nanoparticles (Figure 7 and Figure 8), were assessed by evaluating the bacterial growth as a function of time at two different concentrations (20 μM and 40 μM). More specifically, the bacterial growth was monitored over time by measuring optical density at 600 nm (OD_600_) (Figure 9). OD_600_ was used as a comparative, time-resolved readout of culture dynamics rather than as an absolute measure of viable cell number. Empty CS-PAL nanoparticles were included in parallel as carrier controls to account for potential optical contributions of the nanoparticle matrix.

A clear concentration-dependent reduction in OD_600_, compared with untreated cells (Figure 9, left panel), was observed at the early growth phase (OD_600_ about 0.15). Treatment with empty CS-PAL nanoparticles was characterized by a modest growth-modulating effect, consistent with previous reports on the intrinsic antibacterial activity of chitosan-based materials.^40–42^ During the exponential growth phase (OD600 about 0.6; Figure 9, right panel), CUR-loaded CS-PAL nanoparticles affected bacterial growth to a greater extent compared to the effect of the carrier alone. This growth-modulating effect is consistent with previously reported intracellular mechanisms of CUR, which is primarily associated with modulation of essential cellular processes, rather than with immediate membrane damage or lytic activity. In other words, OD600 profiles are here interpreted as indicators of altered growth dynamics, rather than direct measures of bacterial viability.^16,20^

### 3.4 Minimum Inhibitory Concentration (MIC) and Minimum Bactericidal Concentration (MBC) Evaluation

MIC and MBC were evaluated using the standard broth microdilution method according to CLSI guidelines (Mueller-Hinton medium; 24 h incubation). MIC is defined as the lowest concentration preventing visible bacterial growth, whereas MBC is defined as the lowest concentration preventing recovery of viable colonies after subculture on drug-free agar plates.

As summarized in Table 3, CUR-loaded CS-PAL nanoparticles exhibited a detectable MIC of 80 μM. To ensure that the observed effect was not influenced by optical interference arising from the nanoparticle formulation, such as light scattering or absorbance effects, empty CS-PAL nanoparticles were included as controls.

**Table 3:** MIC of CUR-Loaded CS-PAL Nanoparticles Against *E. coli* Determined by Broth Microdilution in Mueller–Hinton Medium After 24 h Incubation at 37 °C. Results are Representative of Three Independent Experiments

treatment	CUR eq conc. (μM)	cell growth
CUR-CS-PAL	5	+
CUR-CS-PAL	10	+
CUR-CS-PAL	20	+
CUR-CS-PAL	40	+
CUR-CS-PAL	80	-(MIC)
CUR-CS-PAL	160	-

To determine whether growth inhibition was associated with loss of bacterial viability, qualitative assessment of bacterial recovery and MBC assays were performed under the same experimental conditions. For MBC determination, aliquots from wells showing no visible growth were subcultured onto drug-free agar plates. Recovery of viable colonies was observed in all tested conditions, including at the highest CUR-loaded CS-PAL concentrations. Consistent with the MBC results, qualitative CFU assessment did not reveal an evident impairment of bacterial recovery under the experimental conditions employed, suggesting a predominantly bacteriostatic rather than bactericidal effect. Empty CS-PAL nanoparticles had little effect on bacterial recovery, supporting the conclusion that the observed activity mainly reflects the action of intracellularly delivered curcumin.

The agreement between OD-based growth profiles, MIC/MBC results, and qualitative CFU assessment suggests that CUR-loaded CS-PAL exerts a predominantly bacteriostatic effect rather than a bactericidal one. This interpretation is consistent with previously reported intracellular mechanisms of curcumin, including interference with bacterial cell division and metabolic processes.^16,20^

## 4 Conclusion

A green and sustainable procedure to efficiently incorporate CUR into a CS-based nanosystem was developed, and the obtained nanoformulations proved to represent a promising strategy to overcome the physicochemical limitations of this natural compound. A careful spectroscopic analysis (UV–vis and fluorescence techniques) confirmed the chemical integrity of curcumin delivered by the CS-PAL nanosystem. Several biological experiments demonstrated that effective intracellular delivery is a crucial factor for the antibacterial activity of curcumin in Gram-negative bacteria. In fact, the obtained results demonstrated that, by employing CS-PAL nanoparticles as delivery vehicles, curcumin can be efficiently internalized into *E. coli*. The observed intracellular accumulation is consistent with overcoming permeability barriers and solubility limitations that may restrict the availability of free curcumin. The combined evidence from fluorescence imaging and quantitative lysate analysis supports true intracellular accumulation rather than superficial cell association. Importantly, nanoparticle-mediated delivery of CUR was associated with concentration-dependent effects on bacterial growth dynamics. Future work combining higher-resolution imaging (e.g., confocal or ultrastructural methods) with quantitative analytical approaches may further refine intracellular localization and absolute uptake levels. Although qualitative assessment of bacterial viability was included in the present study, additional orthogonal approaches, such as metabolic activity measurements, may further refine the interpretation of growth modulation.

## Abbreviations

ATR: attenuated total reflectance
CFU: colony-forming unit
CH_3_CN: acetonitrile
CLSI: Clinical and Laboratory Standards Institute
CS: chitosan
CS-FA: Chitosan–fatty acid conjugate
CS-LINO: Chitosan–Linoleate conjugate
CS-OLE: Chitosan–Oleate conjugate
CS-PAL: Chitosan–Palmitate conjugate
CUR: curcumin
CUR-CS-PAL: CUR- loaded CS-PAL
DIC: *N*,*N*′-diisopropylcarbodiimide
DLS: dynamic light scattering
DMSO: dimethyl sulfoxide
EtOH: ethanol
FA: fatty acid
FT-IR: fourier transform infrared spectroscopy
HPLC: high-performance liquid chromatography
LB: Luria–Bertani
MBC: minimum bactericidal concentration
MH: Mueller–Hinton
MIC: minimum inhibitory concentration
MW: microwave
MWCO: molecular weight cut-off
NaCl: sodium chloride
OD600: optical density at 600 nm
PDI: polydispersity index
RC: regenerated cellulose
RFU: relative fluorescence units
RP-HPLC: reversed-phase high-performance liquid chromatography
SEC: size exclusion chromatography
TFA: trifluoroacetic acid
TOF: time-of-flight
UV–vis: ultraviolet–visible spectroscopy
